# Prediction of p53 mutation status in rectal cancer patients based on magnetic resonance imaging-based nomogram: a study of machine learning

**DOI:** 10.1186/s40644-023-00607-1

**Published:** 2023-09-18

**Authors:** Xia Zhong, Jiaxuan Peng, Zhenyu Shu, Qiaowei Song, Dongxue Li

**Affiliations:** 1 The First Clinical Medical College, Zhejiang Chinese Medical University, Hangzhou, Zhejiang China; 2 Jinzhou Medical University, Jinzhou, Liaoning Province China; 3 Cancer Center, Department of Radiology, Zhejiang Provincial People’s Hospital, Affiliated People’s Hospital, Hangzhou Medical College, Hangzhou, Zhejiang China

**Keywords:** Nomogram, Rectal cancer, Machine learning, p53 gene, Magnetic resonance imaging

## Abstract

**Background:**

The current study aimed to construct and validate a magnetic resonance imaging (MRI)-based radiomics nomogram to predict tumor protein p53 gene status in rectal cancer patients using machine learning.

**Methods:**

Clinical and imaging data from 300 rectal cancer patients who underwent radical resections were included in this study, and a total of 166 patients with p53 mutations according to pathology reports were included in these patients. These patients were allocated to the training (n = 210) or validation (n = 90) cohorts (7:3 ratio) according to the examination time. Using the training data set, the radiomic features of primary tumor lesions from T2-weighted images (T2WI) of each patient were analyzed by dimensionality reduction. Multivariate logistic regression was used to screen predictive features, which were combined with a radiomics model to construct a nomogram to predict p53 gene status. The accuracy and reliability of the nomograms were assessed in both training and validation data sets using receiver operating characteristic (ROC) curves.

**Results:**

Using the radiomics model with the training and validation cohorts, the diagnostic efficacies were 0.828 and 0.795, the sensitivities were 0.825 and 0.891, and the specificities were 0.722 and 0.659, respectively. Using the nomogram with the training and validation data sets, the diagnostic efficacies were 0.86 and 0.847, the sensitivities were 0.758 and 0.869, and the specificities were 0.833 and 0.75, respectively.

**Conclusions:**

The radiomics nomogram based on machine learning was able to predict p53 gene status and facilitate preoperative molecular-based pathological diagnoses.

**Supplementary Information:**

The online version contains supplementary material available at 10.1186/s40644-023-00607-1.

## Background

Rectal cancer is one of the most common digestive tract malignancies [1], with approximately 1.4 million new diagnoses and 700,000 deaths worldwide each year. The incidence of rectal cancer has been reported as high as 6%, and the five-year survival rate is only 40–60% [2]. Molecular features of rectal cancer related to the tumor microenvironment, such as extramural venous invasion (EMVI), tumor protein p53, and cell proliferation antigen Ki67 levels, are of great interest due to their correlation with prognostic indicators, such as tumor aggressiveness and recurrence risk [3].

Wild-type p53 is an important tumor suppressor gene [4]. However, a mutated version of p53 is found in many human cancers [5], and upon gene mutation or deletion, p53 becomes pro-tumorigenic [6]. Previous studies have shown that p53 plays a role in predicting the effectiveness of neoadjuvant therapy for rectal cancer [7]. In addition, there is a significant correlation between the mutation status of p53 according to gene sequencing analysis and overall survival as well as metastasis-free survival of patients with rectal cancer. Therefore, evaluating p53 gene status is important to accurately determine the prognosis of patients with colorectal cancer [8]. Unfortunately, determining the p53 gene status depends primarily on immunohistochemistry, which limits the prevalence of its detection.

Currently, magnetic resonance imaging (MRI) has the advantage of high soft tissue resolution, and it has become the first choice for early overall non-invasive evaluation for preoperative staging of rectal cancer [9, 10]. Importantly, there have been some tentative studies that also use MRI to assess p53 gene status. For example, Li et al. found that high expression of p53 was correlated with a low tumor enhancement/T2 ratio [11]. In addition, another study revealed that magnetic resonance spectroscopy (MRS) and diffusion weighted imaging (DWI) may be able to accurately reflect p53 status [12], but a visual evaluation of these images may not be enough to identify p53 gene status. Therefore, an objective and non-invasive method to accurately evaluate p53 gene status prior to surgery is still required.

Radiomics is a relatively new field that uses emerging technologies to extract features from medical imaging, quantifying its phenotypic characteristics in a high-throughput manner [13]. Such features may help in prognosticating, predicting treatment outcomes, and assessing tissue malignancy in cancer research [14, 15]. Previous studies have shown that MRI-based radiomics can be used to evaluate the gene status of p53 in gliomas [16]. However, there have not been any studies using MRI to predict p53 gene status in rectal cancer. In addition, previous studies have shown that machine learning models can more accurately predict p53 gene mutations in low-grade gliomas and pancreatic cancer [16, 17].

Therefore, this study aimed to use machine learning to build a radiomics signature that could predict p53 gene status in rectal cancer. The radiomics signature could then be combined with clinical features to build a visualized nomogram to evaluate p53 gene status.

## Materials and methods

### Patient data

This retrospective study was approved by the Ethics Committee of Zhejiang Provincial People’s Hospital and informed consent was obtained. A total of 1056 rectal cancer patients in the picture archiving and communication system with a definitive clinical diagnosis between February 2018 and May 2022 were identified. Next, the clinical and radiology data from 300 patients were selected for retrospective analysis according to the inclusion and exclusion criteria. The inclusion criteria were: (1) diagnosis of rectal cancer by pathological examination; (2) lack of immunotherapy or neoadjuvant chemoradiotherapy; and (3) surgery was performed within one month after examination by MRI. The exclusion criteria were: (1) poor MRI quality or the lesions were poorly displayed; (2) lack of pathological results upon resection. All patients were allocated at a 7:3 ratio to either the training (n = 210) or validation (n = 90) cohort according to when their examination was completed (i.e.: the training group was filled first, then the validation group). The training data set was used to build the radiomics model, and the validation data set was used to verify the performance of the model.

### Immunohistochemistry of p53

Paraffin-embedded tissue sections were deparaffinized in a series of gradient ethanol baths, rehydrated, and immersed in methanol containing 0.3% hydrogen peroxide for 10 min to block endogenous peroxidase at room temperature. Subsequently, the tissue-slides were heated for 30 min in a pH 6.0 antigen retrieval solution to induce antigen retrieval and then incubated overnight with an anti-P53 antibody at 4 °C. Staining was performed using a Prolink-2 Plus HRP rabbit polymer detection kit (Golden Bridge, Bothell, WA, USA) according to the manufacturer’s instructions. Images were captured using Aperio ScanScope CS software (Aperio Technologies, Vista, CA, USA).

The results were evaluated based on the intensity and extent of staining by two independent pathologists (double blinded) as described previously. Briefly, the P53 positive staining area was scored as follows: the staining percentage of positively stained area over total tissue area was defined as 0 = ≤ 5%; 1 = 5–25%; 2 = 26–50%; 3 = 51–75%; and 4 ≥ 75%. The intensity was graded as follows: 0, negative; 1+, weak (yellow); 2+, moderate (light brown); and 3+, strong (dark brown). A final score between 0 and 12 was achieved by multiplication of the staining area and intensity scores. A staining index was used in which a score of 0–2 was considered negative expression, 3–6 was considered low expression, and ≥ 6 was considered high expression.

### Evaluation of baseline clinical characteristics

The clinical and histopathological data of all patients were reviewed and recorded from the patients’ electronic medical records, and included sex, age, carcinoembryonic antigen (CEA) levels (abnormal: > 5 ng/mL), and pathological p53 gene status. The clinical imaging features were obtained by consulting the structured MRI rectal examination report, and included tumor node metastasis (TNM) staging, distance from the end of the convex edge of the tumor to the edge of the anus (DIS), circumferential resection margin (CRM) status, and presence of EMVI. A positive CRM status was defined as tumor, metastases, metastatic lymph nodes, or intramuscular vascular invasion within 1 mm of the mesenteric fascia. EMVI was defined as (a) presence of tumor signal intensity within a vascular structure, (b) expanded vessels, (c) tumoral expansion through and beyond the vessel wall, and (d) disrupting the vessel border.

All data were assessed independently by two experienced radiologists in a double-blind setting. Radiologist A had three years of experience and Radiologist B had ten years of experience in abdominal imaging. Consensus was reached in all cases of disagreement after all assessments were completed.

### Image pre-processing

All patients underwent rectal MRI in a supine position using 3.0T MRI systems (Skyra; Siemens Healthineers) equipped with an eight-channel phased-array coil. In this study, we only used T2-weighted images (T2WI) to extract radiomics features. Not only do T2WI provide clear images of tumor structures that can easily delineate regions of interest, but their high spatial resolution also reduces the impact of image quality on the extracted radiomics features. Export T2WI data for each patient from the Picture Archiving and Communication System (PACS), and then import it into non-commercial Artificial Intelligence Kit software (AK, GE Healthcare, China) for preprocessing of T2WI. Image preprocessing was performed by resampling the images with a resolution of 1 × 1 × 1 mm3 using the linear interpolation method. The image gray level was discretized and normalized to order 32. Next, the preprocessed T2WI were imported into ITK-SNAP software (www.itksnap.org) to segment the entire rectal tumor layer by layer to obtain the volume of interest (VOI) [18]. Blood vessels and necrotic tissues were avoided. Finally, the VOI was imported into the AK software for feature extraction.

### Acquisition and selection of radiomics features

A total of 930 features were extracted from the T2WI of each patient, and specific feature information can be found in Supplementary Materials Table S1. To ensure the stability and accuracy of features extracted from the VOIs, the two radiologists who previously evaluated the clinical features manually and independently performed the tumor segmentation. Feature set A and feature set B were obtained from radiologist A and radiologist B, respectively. Spearman rank correlation was used to determine the correlation coefficient (CC) of each feature between set A and set B. The features with a CC > 0.8 were considered robust.

Optimal features among the robust features were obtained by dimensionality reduction based on the training set. Firstly, analysis of variance was used to calculate the variance of each feature. The variance value is calculated as the average of the squared differences between each variable’s value and the mean. It is the most important method for measuring the dispersion of numerical data. The larger the variance, the greater the fluctuation of the data, and vice versa. So, it is necessary to eliminate features with a variance of 0 or less as a priority. In this study, we calculated the variance of each feature and retained the features that were greater than the threshold of 1. Secondly, minimum redundancy maximum relevance (mRMR) was used to extract optimal features. The purpose of mRMR was to select features that have the greatest correlation with the pathological p53 gene status. The radiomics features with an inter class CCs > 0.8 were retained. Subsequently, to ensure minimal redundancy, intra-class correlation analysis was performed on the remaining features, retaining features with an intra class CCs ≤ 0.1. Finally, the gradient boosting decision tree (GBDT) algorithm was used to reduce the dimension of the remaining features.

### Nomogram construction and performance evaluation

**Fig. 1:**
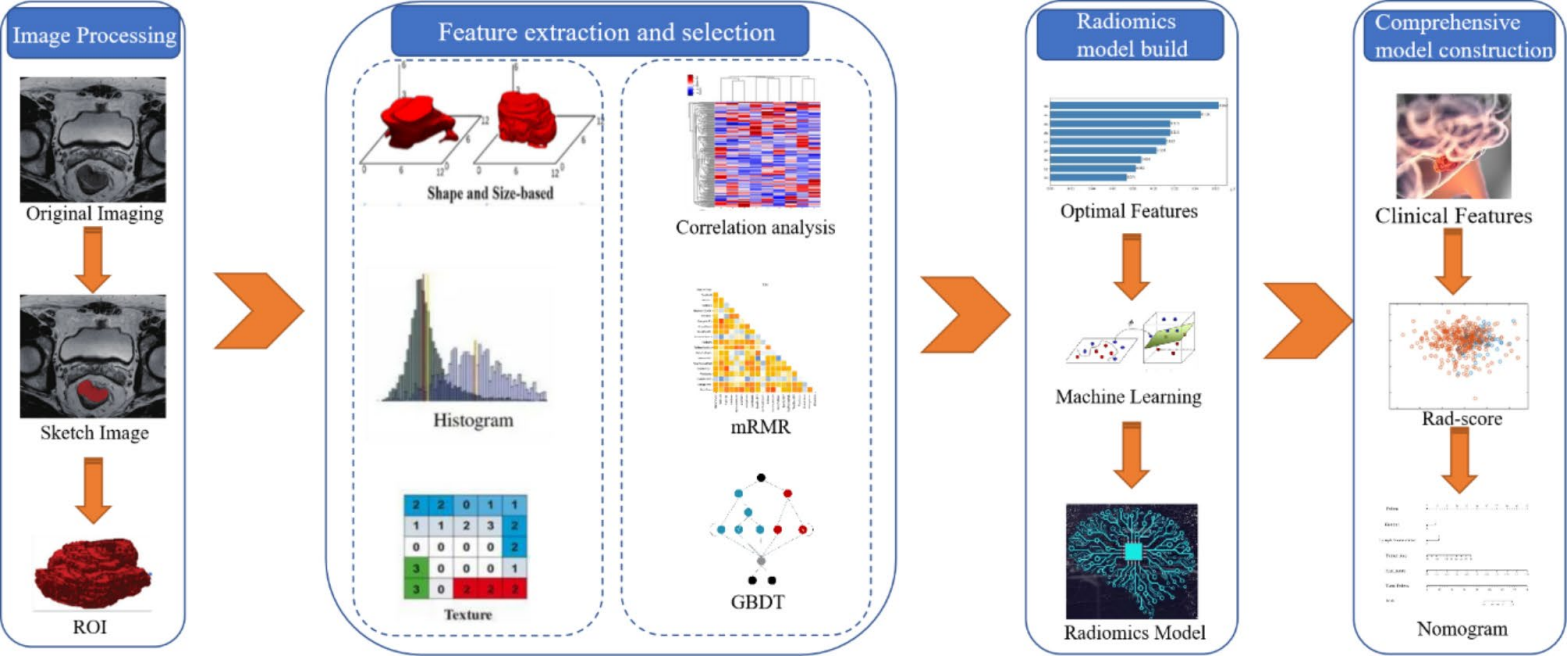
The radiomics workflow

A support-vector machine (SVM) was used to build the signature model based on the final screened features. Ten-fold cross-validation involved stratifying and dividing the training data set into ten folds of equal size: eight folds (80%) for training, one fold (10%) for tuning model parameters, and one fold (10%) for testing. Stratification was used to ensure a similar distribution of events across the ten folds. This process was repeated for ten iterations, always using a different data fold for signature model training, tuning, and testing. The data from the training subgroup and the data from the tuning model parameters subgroup were used to construct a model, resulting in 100 models. Finally, the data from the test subgroup was selected to test the performance of the model. The SVM parameters of the model with the highest accuracy were then chosen as the final tuning parameters. In this study, SVM with a Gaussian kernel function were implemented. The cost parameter C was varied with values{2^− 2^, 2^− 1^, 1, 2^1^, 2^2^} and the kernel spread parameter was varied with values in {10^− 2^, 10^− 1^, 1, 10^1^, 10^2^}. The machine learning scores were concatenated from all testing data folds to assess the signature model performance over the entire data set. The signature model quantified the discriminability as the possibility of a p53 mutation for each patient defined as Rad-score. ROC curves were used to evaluate the performance of the signature model, and the validation data set was used for verification. Next, independent predictors from clinical features and the rad-score were determined using multiple factor logistic regression with a backward stepwise selection method. This method ensured that only significant variables were included in the regression equation. The process continued until no significant explanatory variables were added to the equation and less significant variables were no longer eliminated. Akaike information criterion was used to evaluate the models. A integrated model was then established and a visual nomogram was constructed. Finally, the difference between the integrated model and the signature model was evaluated using the DeLong test, and the nomogram goodness-of-fit was assessed using the Hosmer-Lemeshow test. The radiomics workflow was shown in Fig. 1.

### Statistical analysis

Statistical analyses were performed using MedCalc software (version 11.2), Python (version 3.5), R software (version 3.4.1) and SPSS (version 24.0). Continuous variables were compared by independent sample *t*-test or Mann–Whitney *U* test, and categorical variables were compared using a chi-square test. All statistics were two-sided, and statistical significance was set at *P* < 0.05.

## Results

### Baseline demographic and clinical characteristics of patients

There was no statistical difference in demographic characteristics or conventional radiology characteristics between the training and validation sets (all *P* > 0.05), as shown in Table 1. Additionally, there was no statistical difference in demographic characteristics and conventional radiology characteristics between mutant and wild-type p53 genes in either the training or validation data sets (all *P* > 0.05), as shown in Table 2.

**Table 1:** Clinical features of patients in training and validation cohorts

Characteristics		Training cohort (n = 210)	Validation cohort (n = 90)	P-value
Gender (n, %)	Male	148 (70.48)	64 (71.11)	0.912
	Female	62 (29.52)	26 (28.89)	
CRM status (n, %)	Negative	161 (76.67)	67 (74.44)	0.68
	Positive	49 (23.33)	23 (25.56)	
Mri-EMVI status (n, %)	Negative	161 (76.67)	64 (71.11)	0.309
	Positive	49 (23.33)	26 (28.89)	
ACI status (n, %)	Negative	201 (95.71)	83 (92.22)	0.34
	Positive	9 (4.29)	7 (7.78)	
Lymph Node (n, %)	No metastasis	78 (37.14)	33 (36.67%	0.938
	Metastasis	132 (62.86)	57 (63.33)	
Tumor stage (n, %)	T1-T2	63 (30.00)	17 (18.89)	0.046
	T3-T4	147 (70.00)	73 (81.11)	
Age (years)		64.19 ± 10.47	64.13 ± 9.73	0.965
CEA (ng/mL)		10.13 ± 39.74	12.63 ± 37.67	0.613
Tumor size (cm^3^)		17.63 ± 20.04	21.63 ± 32.62	0.195
Dis (cm)		8.02 ± 3.77	7.98 ± 3.65	0.934

Abbreviations: CRM, circumferential resection margin; EMVI, extramural vascular invasion; ACI, anal canal invasion; CEA, carcinoembryonic antigen; Dis, distance from the end of the convex edge of the tumor to the edge of the anus. Data were presented as counts with percentages or means ± standard deviations

**Table 2:** Baseline features of p53 status in the training and validation cohorts

Characteristics		Training cohort (n = 210)			Validation cohort (n = 90)		
		p53 WT (n = 90)	p53 Mut (n = 120)	P- value	p53 WT (n = 44)	p53 Mut (n = 46)	P- value
Gender (n, %)	Male	57 (63.33)	91 (75.83)	0.066	33 (75.00)	31 (67.39)	0.426
	Female	33 (36.67)	29 (24.17)		11 (25.00)	15 (32.61)	
CRM status (n, %)	Negative	70 (77.78)	91 (75.83)	0.742	30 (68.18)	37 (80.43)	0.183
	Positive	20 (22.22)	29 (24.17)		14 (31.82)	9 (19.57)	
Mri-EMVI status (n, %)	Negative	71 (78.89)	90 (75.00)	0.51	33 (75.00)	31 (67.39)	0.426
	Positive	19 (21.11)	30 (25.00)		11 (25.00)	15 (32.61)	
ACI status (n, %)	Negative	86 (95.56)	115 (95.83)	0.806	38 (86.36)	45 (97.83)	0.102
	Positive	4 (4.44)	5 (4.17)		6 (13.64)	1 (2.17)	
Lymph Node (n, %)	No met	38 (42.22)	40 (33.33)	0.187	16 (36.36)	17 (36.96)	0.953
	Met	52 (57.78)	80 (66.67)		28 (63.64)	29 (63.04)	
Tumor stage (n, %)	T1-T2	23 (25.56)	40 (33.33)	0.224	10 (22.73)	7 (15.22)	0.363
	T3-T4	67 (74.44)	80 (66.67)		34 (77.27)	39 (84.78)	
Age (years)		63.57 ± 11.23	64.66 ± 9.89	0.456	64.50 ± 9.36	63.78 ± 10.17	0.729
CEA (ng/mL)		6.41 ± 8.46	12.92 ± 51.98	0.241	17.33 ± 52.44	8.13 ± 11.69	0.249
Tumor size (cm^3^)		19.79 ± 24.61	16.00 ± 15.70	0.175	22.86 ± 30.02	20.45 ± 35.23	0.728
Dis (cm)		8.27 ± 3.91	7.83 ± 3.67	0.397	7.63 ± 3.80	8.31 ± 3.52	0.382

Abbreviations: WT, wild-type; Mut, mutant; CRM, circumferential resection margin; EMVI, extramural vascular invasion; ACI, anal canal invasion; CEA, carcinoembryonic antigen; Dis, distance from the end of the convex edge of the tumor to the edge of the anus. Data were presented as counts with percentages or means ± standard deviations

### Radiomics feature selection

**Fig. 2:**
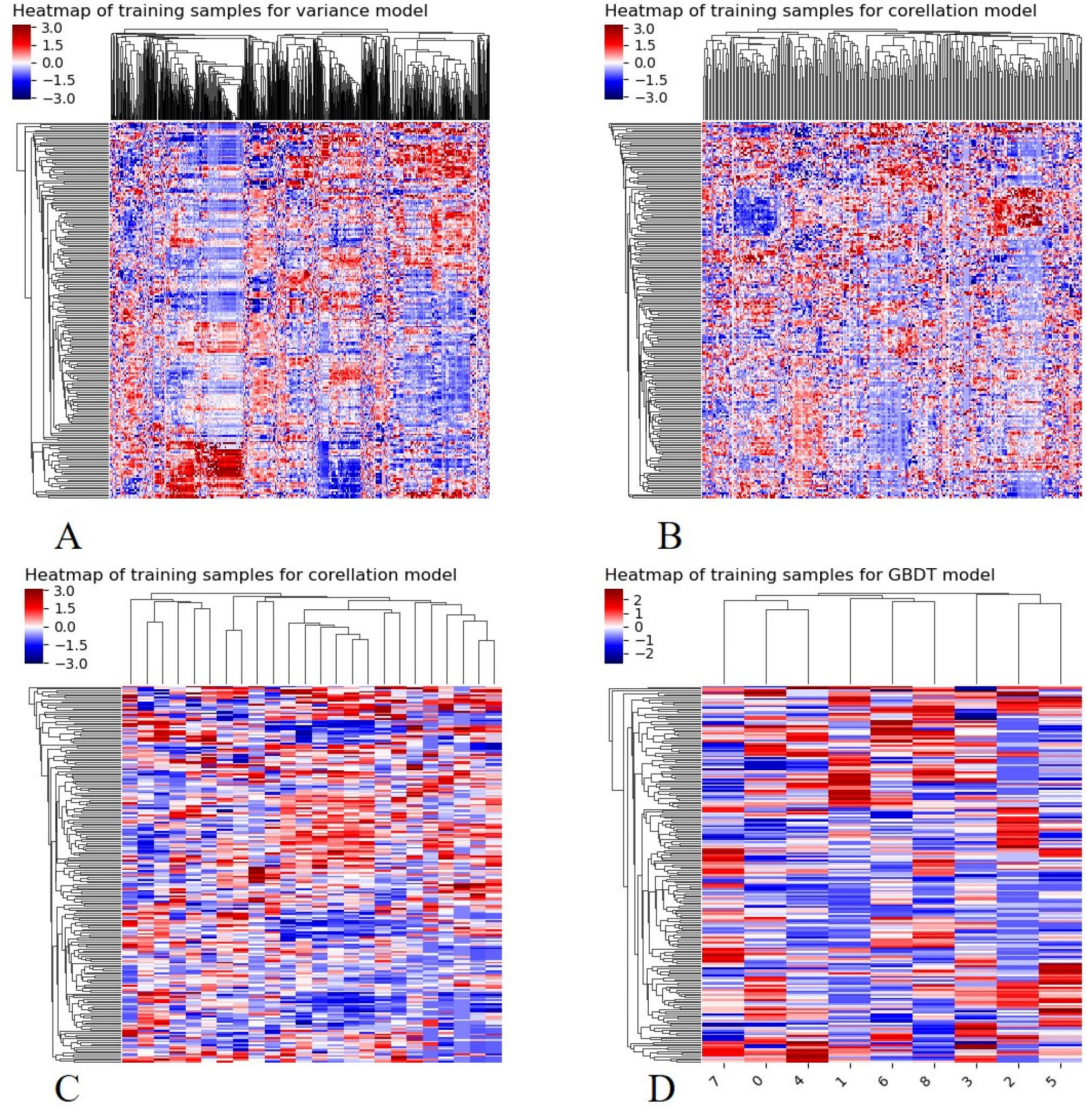
Process of heat map for dimension reduction. The x-axis represents feature ordering, the y-axis represents 300 case sequencing, and the color represents feature value size. (**A**) Features selected based on analysis of variance, and the X-axis is 874 features. (**B**) Features extracted from the correlation analysis with clinical outcomes, The X-axis has 476 features. (**C**) Features extracted from the correlation analysis between features, The X-axis number is 24 features. (**D**) The remaining features after dimensionality reduction using a gradient boosted decision tree (GBDT), The X-axis number is 9 features

**Fig. 3:**
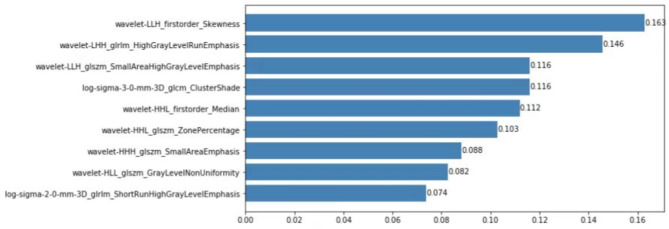
The weight diagram of the final nine features used to build the radiomics model. The x-axis represents the weight or contribution of each feature that is associated with a p53 mutation. The larger the value, the greater its contribution to the model, and the value behind each blue column represents the weight value of this feature in the model

A total of 930 features were extracted from the images, and 912 of these were determined to be robust (CC > 0.8). With further screening, 874 features were obtained based on the variance results. After mRMR dimensionality reduction, 24 features were identified, and finally nine features remained after GBDT dimensionality reduction (Fig. 2). These nine features listed in Fig. 3 were used to build the radiomics model.

### Nomogram model construction and evaluation

**Fig. 4:**
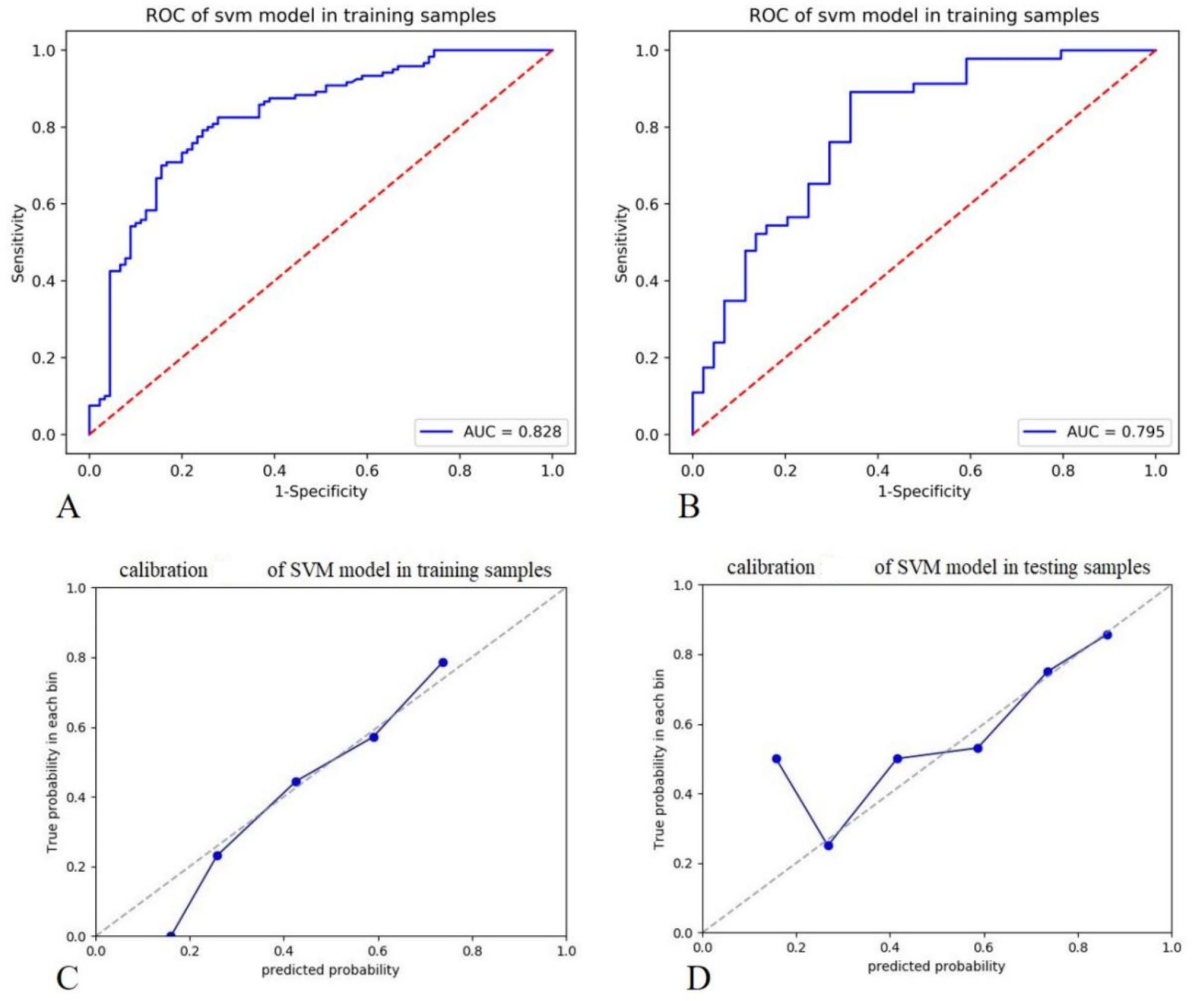
The diagnostic efficiency of the support-vector machine (SVM)-based radiomics signature for predicting p53 mutation status in the training cohort (**A**) and the validation cohort (**B**). The calibration curves of the joint model for predicting p53 mutation status in the training cohort (**C**) and the validation cohort (**D**), which demonstrated good agreement with the ideal curve

**Fig. 5:**
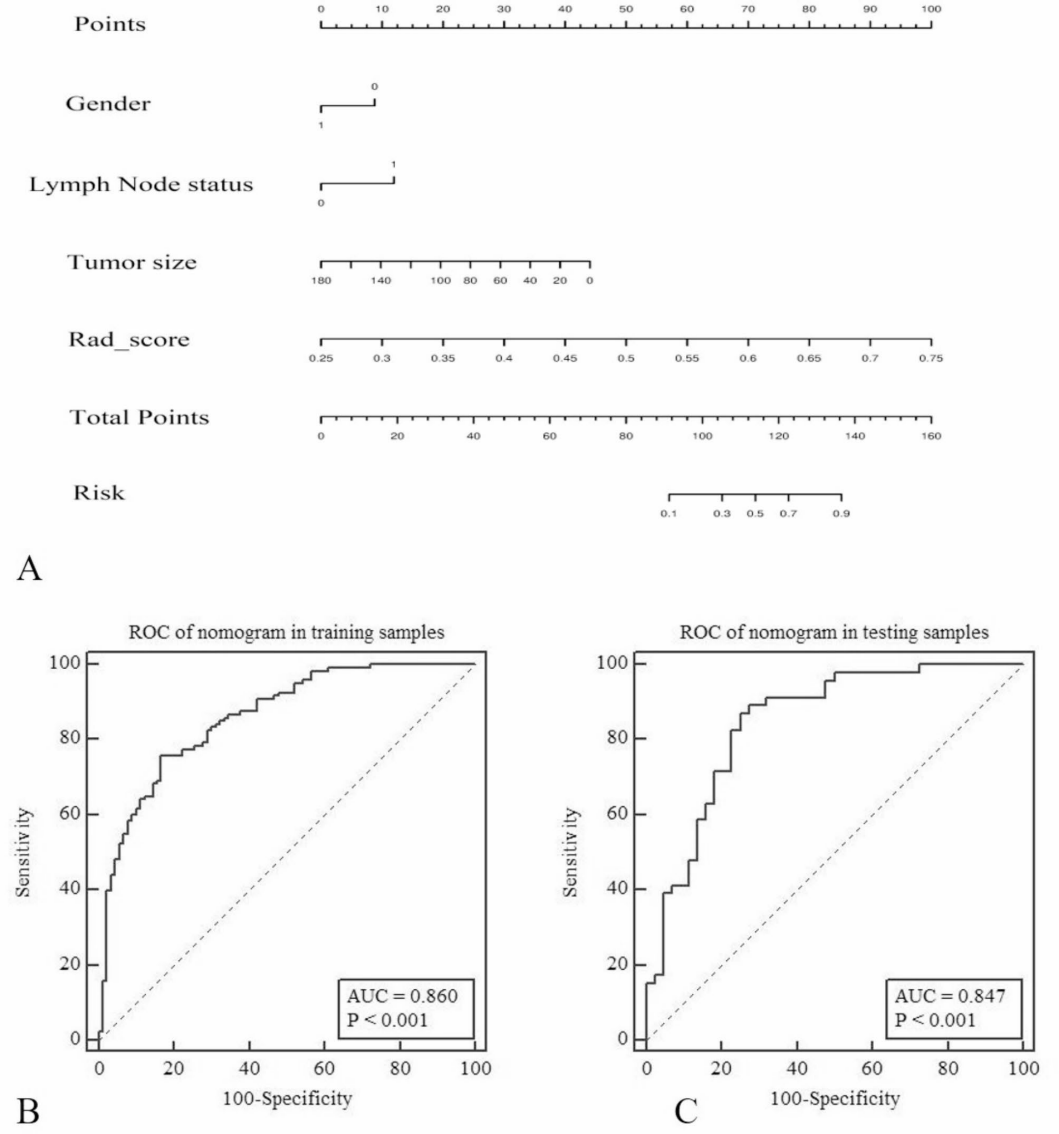
Visual nomogram based on the integrated model (**A**). The diagnostic efficiency of the nomogram for predicting p53 mutation status in the training cohort (**B**) and the validation cohort (**C**)

The area under the curve (AUC) values of receiver operating characteristic curve (ROC) showed that the diagnostic efficacies of the radiomics signature model in the training and validation data sets were 0.828 and 0.795, the sensitivities were 0.825 and 0.891, and the specificities were 0.722 and 0.659, respectively. The calibration curves reveal that the predicted p53 gene status of the signature model in both the training and verification groups was in good consistency with the actual p53 gene status, as shown in Fig. 4. Multivariate logistic regression showed that sex, lymph node metastasis, tumor volume, and rad-scores were independent predictors of p53 gene status (Table 3). The diagnostic efficacies of the nomogram in the training and validation data sets were 0.86 and 0.847, the sensitivities were 0.758 and 0.869, and the specificities were 0.833 and 0.75, respectively, as shown in Fig. 5. For the nomogram, the results of a Hosmer-Lemeshow test suggested no significant deviation (*P* = 0.123) from an ideal fitting.

**Table 3:** Independent predictors of p53 status by multivariate logistic regression analysis

Variable	Univariate logistic regression		Multivariate logistic regression	
	OR (95%CI)	P-value	OR (95%CI)	P-value
Sex	0.361 (0.164,0.796)	0.011*	0.359 (0.166,0.776)	0.009*
CRM status	0.672 (0.248, 1.818)	0.434	NA	NA
Mri-EMVI status	2.435 (0.846, 7.01)	0.099	NA	NA
ACI status	0.669 (0.109, 4.099)	0.664	NA	NA
Lymph Node	2.615 (1.114, 6.136)	0.027*	2.42 (1.143,5.121)	0.021*
Tumor stage	0.702 (0.293, 1.682)	0.427	NA	NA
Age	1.005 (0.97, 1.041)	0.788	NA	NA
CEA	1.000 (0.99, 1.011)	0.982	NA	NA
Tumor size	0.976 (0.958, 0.995)	0.012*	0.978 (0.961,0.995)	0.012*
Dis	0.962 (0.871, 1.062)	0.442	NA	NA
Radiomics model score	2.484 (1.104, 11.044)	< 0.001*	1.566 (0.848,2.894)	< 0.001*

Abbreviations: OR, odds ratio; CI, confidence interval; CRM, circumferential resection margin; EMVI, extramural vascular invasion; ACI, anal canal invasion; CEA, carcinoembryonic antigen; Dis, distance from the end of the convex edge of the tumor to the edge of the anus

## Discussion

The results of this study showed that a radiomics signature based on features extracted from T2WI combined with machine learning can be used to predict p53 gene status in patients with rectal cancer. In addition, a visual nomograph was created to ensure that the results of the prediction model are easy to comprehend and convenient for clinicians to evaluate the p53 gene status of rectal cancer patients. As such, this study provides a new tool for pathological molecular diagnoses.

p53 is an important tumor suppressor gene, and its mutation status may be an important factor for early diagnosis and treatment of rectal cancer [19]. However, using molecular biology techniques to detect p53 gene expression and mutations requires substantial time and money, so alternative cost- and time-effective imaging methods are preferred when possible. One previous study confirmed that skewness of magnetic resonance apparent diffusion coefficient histogram analysis parameters was related to p53 gene status in rectal cancer. In the current study, conventional T2WI were used to predict p53 gene status, and the AUC values concerning diagnostic efficacies of the nomogram in the training and validation sets were 0.86 and 0.847, respectively. One heavily weighted feature of the nomogram was the first-order skewness value, which further confirms that conventional MRI images contain information suggestive of p53 gene status. As a conceptual design, this study may also provide a useful tool for screening high-risk populations with P53 mutation in clinical practice.

Previously, both computed tomography (CT)- and MRI-based radiomics have been used to predict p53 status in different cancers. Iwatate et al. established a CT-based radiologic model to predict p53 status in pancreatic cancer [17], while Sun et al. established an MR-based radiologic model to predict p53 status in gliomas [20]. Lin et al. extracted radiologic features from T1-weighted sagittal dynamic contrast-enhanced MRI to predict changes in the p53 mutation status in breast cancer [21]. Although these studies have confirmed the accuracy of radiomics models in predicting p53 status in tumors, it should be noted that all of these studies were based on CT imaging, while our current study employed MRI to predict p53 mutation status in rectal cancer. In comparison to CT, MRI offers advantages such as high resolution of soft tissue and no ionizing radiation. Importantly, our study used non-enhanced MRI images, which eliminates the potential risks associated with contrast agents used in dynamic enhanced MRI.

The results of the current study also showed that tumor size, lymph node metastasis, sex, and the radiomics signature were independent predictive factors of the p53 mutation status. Xu et al. determined that tumor size is the primary radiomic prognostic and predictive feature of microvascular invasion in hepatocellular carcinoma and is independently associated with disease recurrence and mortality [22]. Zhang et al. found that p53 status was positively related to lymph node metastasis in lung cancer [23], which further confirmed that p53 status might be closely related to lymph node metastasis in rectal cancer. However, it should be noted that Pan et al. showed that p53 gene mutations in rectal cancer had no correlation with tumor size or lymph node metastasis [24]. We suspect that this discrepancy may be due to the differences in sample size, since the Pan et al. study only included 97 cases, which is far less than the number of cases assessed in the current study. In addition, a separate study found that gender was important to include in a predictive model for bladder cancer recurrence [25]. Gasinska et al. found that a predictor of long-term overall survival to be male rectal cancer patients negative for p53 [26]. A study by Rockwell et al. discovered that in patients with a glioblastoma, p53 mutations showed sex differences in frequency [27]. The analysis of three p53 genes with repeated mutations revealed a unique correlation between sex and p53 gene mutations using male and female primary mouse astrocytes. Together, these results suggest a possible association between gender and p53 gene status.

In this study, SVM was used to construct the prediction signature. The kernel function in SVM maps the input parameters to different feature spaces, which can divide the transformed data to achieve more accurate results, improve the robustness of the model, and avoid over fitting in the training process. Especially for research with limited cases, SVM is considered the best choice to balance the variance and bias of input data [28–30]. Wang et al. used clinical and CT-based radiomics multiparameter methods to predict p53 gene expression in patients with giant cell tumors of the spine, and the results revealed that the SVM model performed well (AUC = 0.79) [31]. The current study also confirmed this, as the prediction model constructed using SVM could distinguish the p53 gene status in rectal cancer patients (training set, AUC = 0.828; validation set, AUC = 0.795).

There are some limitations in the current study. Firstly, this was a retrospective study and patients were not randomized, which may result in selection bias. Secondly, p53 gene status was confirmed using immunohistochemistry rather than genetic sequencing. However, as a conceptual study, this did not affect the final research outcome. Finally, the study data was collected from a single center, and further multi-center studies are needed to validate the results.

## Conclusions

In conclusion, radiomic features can be used to identify the p53 gene status in cancer, and the nomogram visualization based on this model can serve as a conceptual design for screening high-risk populations with P53 gene status in clinical practice. This may facilitate the pathological molecular diagnosis and risk stratification of locally advanced rectal cancer. In the future, we hope to further validate the model through multidimensional verification in order to expand the detection methods of P53 gene status.

### Electronic supplementary material

Below is the link to the electronic supplementary material.

Supplementary Material 1

## Data Availability

Data are available on reasonable request via the corresponding author.

## Abbreviations

ROC: Receiver Operating Characteristic
EMVI: Extramural Venous Invasion
MRS: Magnetic Resonance Spectroscopy
CEA: Carcinoembryonic Antigen
TNM: Tumor Node Metastasis
CRM: Circumferential Resection Margin
VOI: Volume of Interest
CC: Correlation Coefficient
mRMR: minimum Redundancy Maximum Relevance
GBDT: Gradient Boosted Decision Tree
SVM: Support Vector Machine
AUC: Area under the Curve
