# Supplementary material for: Prediction of p53 mutation status in rectal cancer patients based on magnetic resonance imaging-based nomogram: a study of machine learning
Source: Cancer Imaging. 2023 Sep 18;23:88. doi: 10.1186/s40644-023-00607-1 (PMC10507842; doi:10.1186/s40644-023-00607-1)
Supplement: Supplementary file 1 — Supplementary Material 1 [file 40644_2023_607_MOESM1_ESM.docx]

**Supplementary material**

1. **Standardization of data**

The extracted features were standardized before dimension reduction, which removed the unit limits of the data of each feature and converted it into a dimensionless pure value. This allowed the indices of different units or orders to be compared and weighted. We used Z-score normalization to make the feature intensities fit a standard normal distribution with μ and σ, wherein μ is the mean value of the features and σ is the standard deviation. The normalized values (also called Z-scores) of the feature intensities (x) were calculated as follows:


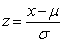


**Table S1. The information of radiomics features**

| Feature Groups (N) | Feature names | Feature Groups (N) | Feature names |
| --- | --- | --- | --- |
|  | firstorder_10Percentile |  | glszm_GrayLevelNonUniformity |
|  | firstorder_90Percentile |  | glszm_GrayLevelNonUniformityNormalized |
|  | firstorder_Energy |  | glszm_GrayLevelVariance |
|  | firstorder_Entropy |  | glszm_HighGrayLevelZoneEmphasis |
|  | firstorder_InterquartileRange |  | glszm_LargeAreaEmphasis |
|  | firstorder_Kurtosis |  | glszm_LargeAreaHighGrayLevelEmphasis |
|  | firstorder_Maximum |  | glszm_LargeAreaLowGrayLevelEmphasis |
| First-order features (N = 18) | firstorder_MeanAbsoluteDeviation | GLSZM texture features | glszm_LowGrayLevelZoneEmphasis |
|  | firstorder_Mean | (N = 16) | glszm_SizeZoneNonUniformity |
|  | firstorder_Median |  | glszm_SizeZoneNonUniformityNormalized |
|  | firstorder_Minimum |  | glszm_SmallAreaEmphasis |
|  | firstorder_Range |  | glszm_SmallAreaHighGrayLevelEmphasis |
|  | firstorder_RobustMeanAbsoluteDeviation |  | glszm_SmallAreaLowGrayLevelEmphasis |
|  | firstorder_RootMeanSquared |  | glszm_ZoneEntropy |
|  | firstorder_Skewness |  | glszm_ZonePercentage |
|  | firstorder_TotalEnergy |  | glszm_ZoneVariance |
|  | firstorder_Uniformity |  |  |
|  | glrlm_GrayLevelNonUniformity |  | glcm_Autocorrelation |
|  | glrlm_GrayLevelNonUniformityNormalized |  | glcm_ClusterProminence |
|  | glrlm_GrayLevelVariance |  | glcm_ClusterShade |
|  | glrlm_HighGrayLevelRunEmphasis |  | glcm_ClusterTendency |
|  | glrlm_LongRunEmphasis |  | glcm_Contrast |
| GLRLM texture features | glrlm_LongRunHighGrayLevelEmphasis | GLCM texture features | glcm_Correlation |
| (N = 16) | glrlm_LongRunLowGrayLevelEmphasis | (N = 24) | glcm_DifferenceAverage |
|  | glrlm_LowGrayLevelRunEmphasis |  | glcm_DifferenceEntropy |
|  | glrlm_RunEntropy |  | glcm_DifferenceVariance |
|  | glrlm_RunLengthNonUniformity |  | glcm_Id |
|  | glrlm_RunLengthNonUniformityNormalized |  | glcm_Idm |
|  | glrlm_RunPercentage_T2 |  | glcm_Idmn |
|  | glrlm_RunVariance |  | glcm_Idn |
|  | glrlm_ShortRunEmphasis |  | glcm_Imc1 |
|  | glrlm_ShortRunHighGrayLevelEmphasis |  | glcm_Imc2 |
|  | glrlm_ShortRunLowGrayLevelEmphasis |  | glcm_InverseVariance |
|  |  |  | glcm_JointAverage |
|  |  |  | glcm_JointEnergy |
|  |  |  | glcm_JointEntropy |
|  |  |  | glcm_MCC |
|  |  |  | glcm_MaximumProbability |
|  |  |  | glcm_SumAverage |
|  |  |  | glcm_SumEntropy |
|  |  |  | glcm_SumSquares |
|  | gldm_DependenceEntropy |  | ngtdm_Busyness |
|  | gldm_DependenceNonUniformity |  | ngtdm_Coarseness |
| GLDM texture features | gldm_DependenceNonUniformityNormalized | NGTDM texture features | ngtdm_Complexity |
| (N = 14) | gldm_DependenceVariance | (N = 5) | ngtdm_Contrast |
|  | gldm_GrayLevelNonUniformity |  | ngtdm_Strength |
|  | gldm_GrayLevelVariance |  |  |
|  | gldm_HighGrayLevelEmphasis |  |  |
|  | gldm_LargeDependenceEmphasis |  |  |
|  | gldm_LargeDependenceHighGrayLevelEmphasis |  |  |
|  | gldm_LargeDependenceLowGrayLevelEmphasis |  |  |
|  | gldm_LowGrayLevelEmphasis |  |  |
|  | gldm_SmallDependenceEmphasis |  |  |
|  | gldm_SmallDependenceHighGrayLevelEmphasis |  |  |
|  | gldm_SmallDependenceLowGrayLevelEmphasis |  |  |
| Los features (N = 186) | Log-sigma-1.0，2.0_* (N =186) |  |  |

Note: GLCM, Gray-level co-occurrence matrices; GLRLM, Gray-level run length matrix; GLSZM, Gray-level size zone matrix; GLDM, Gray-level dependence matrix. *The abbreviated representation of feature types

**2.Machine Learning Details**

SVM, with a Gaussian kernel function was implemented using the caret interface and the R package kernlab. The cost parameter C was varied with values {2^-2^, 2^-1^, 1, 2^1^, 2^2^} and the parameter kernel spread was varied with values in {10^-2^, 10^-1^, 1, 10^1^, 10^2^}.
